# 116. Negative Predictive Value of Surveillance Swabs for Third-Generation Cephalosporin-Resistant Enterobacterales in the Intensive Care Unit

**DOI:** 10.1093/ofid/ofaf695.048

**Published:** 2026-01-11

**Authors:** Sima L Sharara, Eili Klein, Kate Dzintars, Pranita Tamma, Sara E Cosgrove

**Affiliations:** Johns Hopkins Hospital, Baltimore, MD; Johns Hopkins School of Medicine, Baltimore, Maryland; The Johns Hopkins Hospital, Baltimore, Maryland; Johns Hopkins University School of Medicine, Baltimore, Maryland; Johns Hopkins School of Medicine, Baltimore, Maryland

## Abstract

**Background:**

While methicillin-resistant *S. aureus* nasal screening is common in U.S. intensive care units (ICUs) and guides empiric antibiotics, rectal surveillance for resistant Gram-negative organisms (GNO) is less common. Rectal swabs that detect third-generation cephalosporin-resistant Enterobacterales (3GCRE) may serve as a phenotypic proxy for resistant GNO, including extended-spectrum β-lactamase-producing Enterobacterales (ESBL-E). However, the extent to which a negative 3GCRE swab reliably predicts the absence of subsequent resistant GNO infection remains unclear.

**Table 1.**
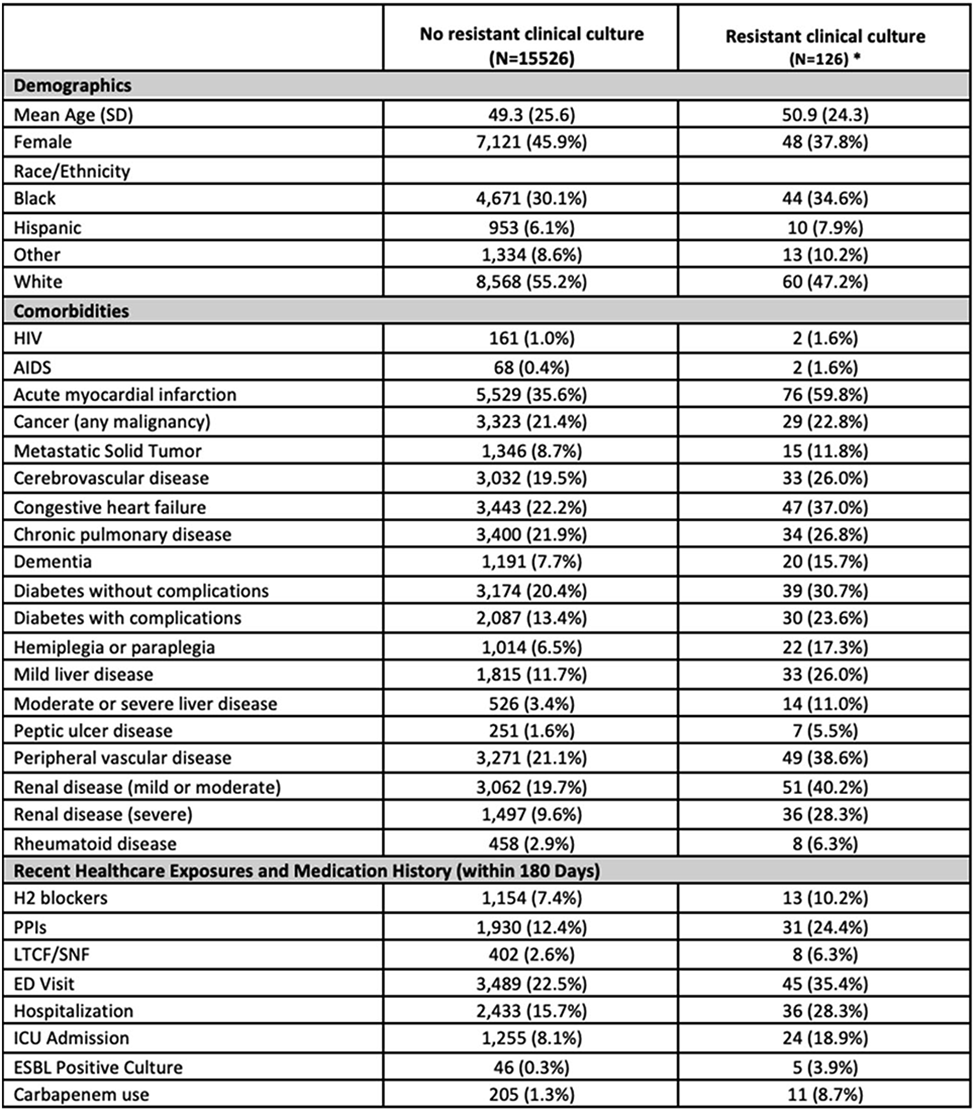
Demographic and Clinical Characteristics of ICU Patients with Negative 3GCRE Surveillance Swabs, Stratified by Presence of a Resistant Clinical Culture During the Same ICU Encounter Abbreviations: LTCF/SNF = long-term care facility/skilled nursing facility; ED = emergency department; ICU = intensive care unit; ESBL = extended-spectrum beta-lactamase; PPI = proton pump inhibitor. * Resistant clinical culture defined as a culture growing a cefepime-intermediate/resistant organism, including extended-spectrum β-lactamase-producing Enterobacterales (ESBL-E) or carbapenemase-producing organisms (CRO) during the ICU stay

**Table 2.**
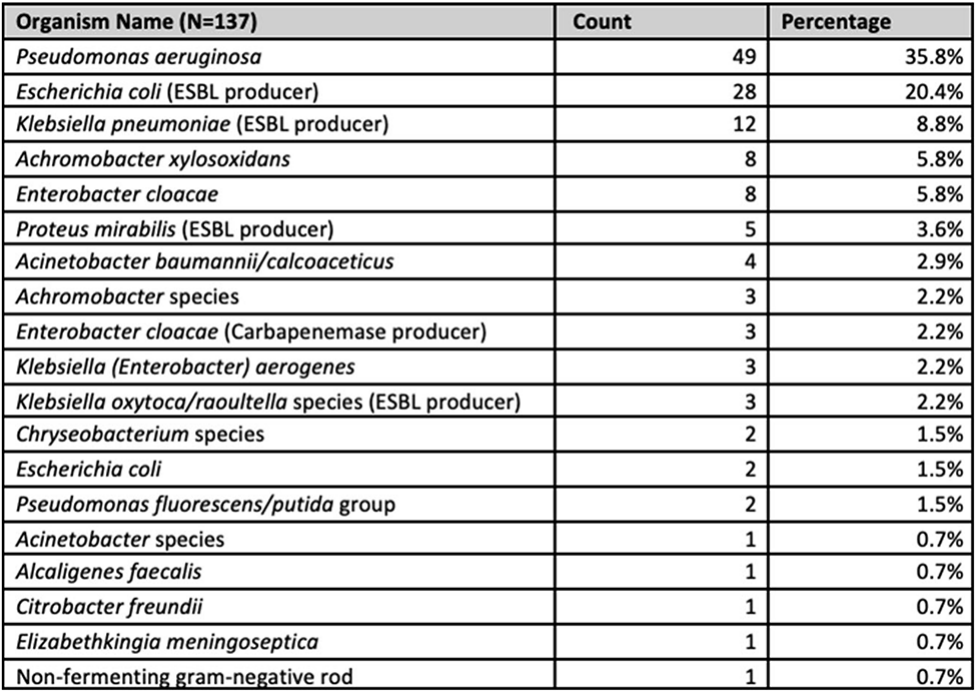
Distribution of Cefepime-Resistant Organisms Identified from Clinical Cultures Among ICU Patients with Negative 3GCRE Surveillance Swabs (N=137) Abbreviations: ESBL = extended-spectrum beta-lactamase

**Methods:**

We conducted a retrospective cohort study of ICU admissions at nine Johns Hopkins Hospital ICUs from July 2022 through April 2025. Rectal surveillance swabs were cultured on HardyCHROM™ ESBL agar to detect 3GCRE. Patients with only negative 3GCRE swabs during their ICU stay were included. Using electronic health record data, we identified patients who subsequently developed clinical cultures growing (1) ESBL-E, (2) carbapenemase-producing organisms (CRO), or (3) cefepime-intermediate or -resistant organisms during the same ICU encounter. Cefepime was assessed as the preferred empiric agent per institutional guidelines.

**Results:**

Among 15,653 patients with negative 3GCRE surveillance swabs, 1,435 (9%) developed a positive clinical culture during their hospital stay. Of these, 127 patients (0.8% of total cohort) had clinical isolates that were cefepime-intermediate or -resistant. Sixty-five (0.4% of total cohort) of these isolates were due to Enterobacterales, 46 (70.8%) were confirmed ESBL-E. Three organisms (2%) were CROs. Commonly identified pathogens were *Pseudomonas aeruginosa*, *Escherichia coli*, and *Klebsiella pneumoniae*. The negative predictive value of a negative 3GCRE swab for detecting cefepime resistance in subsequent clinical isolates was 99.2%.

**Conclusion:**

In this large ICU cohort, a negative 3GCRE swab reliably predicted absence of subsequent infection with resistant GNO. These findings support the use of negative surveillance results 1) to guide early de-escalation of empiric meropenem and 2) avoid starting meropenem if surveillance swabs are negative for 3GCRE. Routine surveillance may help reduce carbapenem use and preserve last-line antibiotic effectiveness in ICUs.

**Disclosures:**

All Authors: No reported disclosures

